# New Real-Time PCRs to Differentiate *Rickettsia* spp. and *Rickettsia conorii*

**DOI:** 10.3390/molecules25194431

**Published:** 2020-09-27

**Authors:** Valeria Blanda, Rosalia D’Agostino, Elisabetta Giudice, Kety Randazzo, Francesco La Russa, Sara Villari, Stefano Vullo, Alessandra Torina

**Affiliations:** 1 Istituto Zooprofilattico Sperimentale della Sicilia, Via Gino Marinuzzi 3, 90100 Palermo, Italy; valeria.blanda@izssicilia.it (V.B.); rosalia.dagostino@izssicilia.it (R.D.); ketyrandazzo@libero.it (K.R.); sara.villari@izssicilia.it (S.V.); stefano.vullo@izssicilia.it (S.V.); alessandra.torina@izssicilia.it (A.T.); 2 Department of Chemical, Biological, Pharmaceutical and Environmental Science, Università degli Studi di Messina, 98122 Messina, Italy; egiudice@unime.it

**Keywords:** *Rickettsia* spp., *R. conorii*, real-time PCR, *ompA*, *ompB*

## Abstract

*Rickettsia* species are an important cause of emerging infectious diseases in people and animals, and rickettsiosis is one of the oldest known vector-borne diseases. Laboratory diagnosis of *Rickettsia* is complex and time-consuming. This study was aimed at developing two quantitative real-time PCRs targeting *ompB* and *ompA* genes for the detection, respectively, of *Rickettsia* spp. and *R. conorii* DNA. Primers were designed following an analysis of *Rickettsia* gene sequences. The assays were optimized using SYBR Green and TaqMan methods and tested for sensitivity and specificity. This study allowed the development of powerful diagnostic methods, able to detect and quantify *Rickettsia* spp. DNA and differentiate *R. conorii* species.

## 1. Introduction

The *Rickettsia* genus (order Rickettsiales; family Rickettsiaceae) includes obligate intracellular, slow-growing Gram-negative bacteria, representing an important cause of emerging vector-borne diseases [1].

These bacteria can be transmitted to animals and humans by blood-sucking arthropods, causing specific zoonotic diseases termed rickettsioses, among the oldest known vector-borne diseases [2,3].

Ticks are the main vectors, although the pathogen can also be transmitted by other arthropods, such as fleas, lice, or mites, with some evidence also for mosquitoes for some *Rickettsia* species (e.g., *Rickettsia felis*) [4,5,6]. The *Rickettsia* genus encompasses several species, and the most widely used classification of *Rickettsia* spp. within this genus includes the spotted fever group (SFG), typhus group (TG), *Rickettsia bellii* group, and *Rickettsia canadensis* group [7].

The SFG *Rickettsia* species comprise two major subgroups: The Rocky Mountain Spotted Fever (RMSF) group, to which *R. rickettsii* belongs, and the Mediterranean Spotted Fever (MSF) group, including *R. conorii* [8].

MSF due to *R. conorii* is endemic in the Mediterranean area, including northern Africa and southern Europe. It has also been described in sub-Saharan Africa, and some cases have also been reported in northern and central Europe [9]. The disease is an emerging or re-emerging disease in some regions, while in some other Mediterranean countries MSF incidence has increased during recent years [10,11]. This could be explained by global warming producing an increase in tick bites [12].

After a tick bite, bacteria replication within the infection site can lead to a necrotic lesion (eschar), and endothelial cells are the first cellular targets for rickettsia infection, with severe vascular damage [13]. MSF symptoms include fever, headache, and maculopapular rash; an eschar at the tick-bite site can also be present [14]. The disease usually has a benign course, but severe complications are reported in about 6% of cases, with patients showing severe vasculitis with renal and other organ involvement and multiorgan failure [4]. Fatal outcomes can occur even in healthy adults, with a 2.5% reported death rate [15,16].

For many years, MSF diseases due to *R. conorii conorii* were thought to be the only tick-borne rickettsial diseases found in southern and eastern Europe. However, in recent years, the amplification and sequencing of different molecular markers has allowed the identification of new *Rickettsia* species or subspecies within the SFG group involved in human rickettsiosis, such as *R. conorii israelensis*, *R. conorii caspia*, *R. aeschlimannii*, *R. slovaca*, *R. sibirica*, *R. mongolitimonae*, and *R.massiliae* [17,18]. These species are considered as emerging pathogens [19], and in many cases they are characterized by distinct clinical features and defined geographic locations [14].

In Italy, SFG rickettsiosis is a mandatory notifiable disease since 1990; nevertheless, due to under-notification and under-diagnosis of cases, the true incidence of the disease remains unknown. As in other Mediterranean countries [20], MSF caused by *R. conorii* is the predominant SFG SFGrickettsiosis. A recently published study estimated a total of 5989 cases of SFG rickettiosis in Italy from 2001 to 2015, with an average annual incidence of 0.88/100000 people and a mean annual standardized hospitalization rate of 1.36/100000 people/year. In particular, cases were strongly concentrated in Sicily and Sardinia, in the southern part of Calabria, and in the southern coastal provinces of the Lazio region [21].

Several methods have been developed for diagnosis of rickettsioses. Serological tests are the easiest methods but data interpretation is often complicated by the cross-reactivity among the species [22]. *Rickettsia* species identification involves molecular methods based on PCR amplification and sequencing of various rickettsial genes; however, the procedure is time-consuming. Real-time PCR (RT-PCR) is a rapid and sensitive method, and quantitative methods based on RT-PCR have been developed for rickettsial agent detection [23,24,25,26].

Genomic approaches have recently increased our knowledge on *Rickettsia* spp., and massive amounts of genomic data have become available. We used published genomic sequence data to design new sets of primers and probes for RT-PCRs targeting the *ompB* and *ompA* genes. These two genes codify for two outer-membrane proteins widely investigated due to their exposed cellular location and the presence of conserved epitopes. Both genes are also commonly employed for phylogenetic analysis due to their significant variability among species [27]. The aim of this study was to improve the diagnosis of *Rickettsia* infections by developing two new RT-PCRs, able to quickly and reliably detect *Rickettsia* spp. DNA and to discriminate, without sequencing, *R. conorii* infection, which is of particular importance since it occurs with high frequency in Mediterranean area, in both humans and animals.

## 2. Results

The newly developed RT-PCR assays showed high sensitivity and specificity. The detection limit of both assays, based on the standard curve dilutions, resulted to be less than 10 DNA copies, while, as regards the conventional PCRs most commonly used for *Rickettsia* species diagnosis [3], we obtained a sensitivity of 100 copies of DNA for single-step PCRs and 10 copies of DNA for nested PCRs (data not shown).

Positive controls were correctly identified. The assays showed good reproducibility when standards were tested in duplicate.

**Figure 1:**
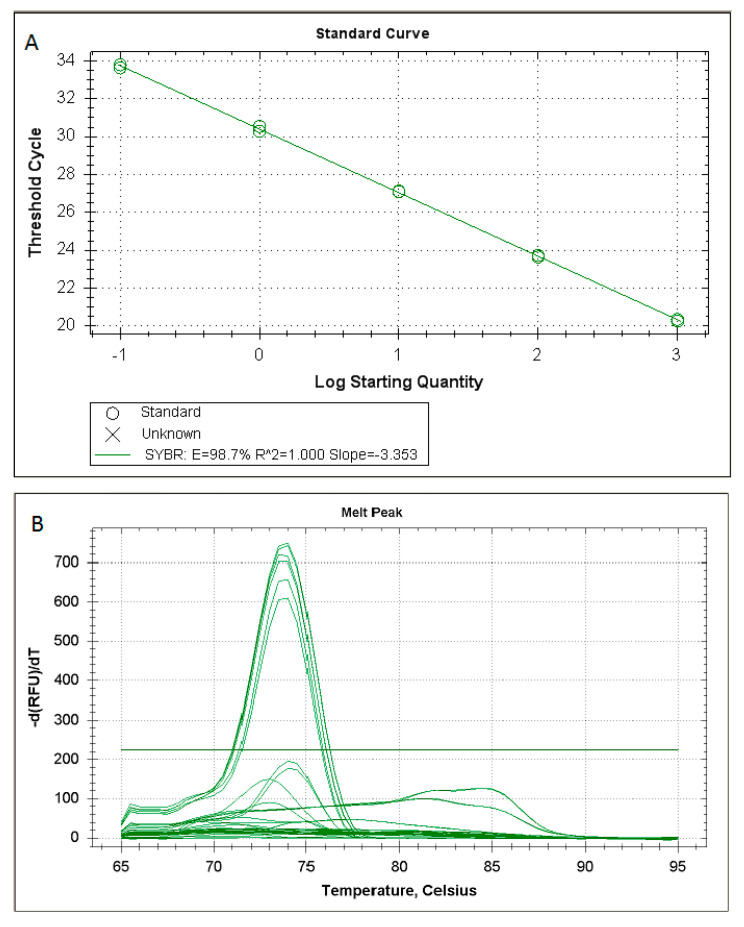
Customary values obtained for the standard curve (**A**) and melting temperature analysis (**B**) of the SYBR Green RT-PCR for *Rickettsia* spp. based on the amplification of an *ompB* fragment.

In particular, Figure 1A shows customary values obtained for the standard curve (r = 1; slope = −3.353) of the SYBR Green RT-PCR for *Rickettsia* spp. based on the amplification of an *ompB* fragment. Melting temperature analysis showed that the reaction was specific (Figure 1B).

**Figure 2:**
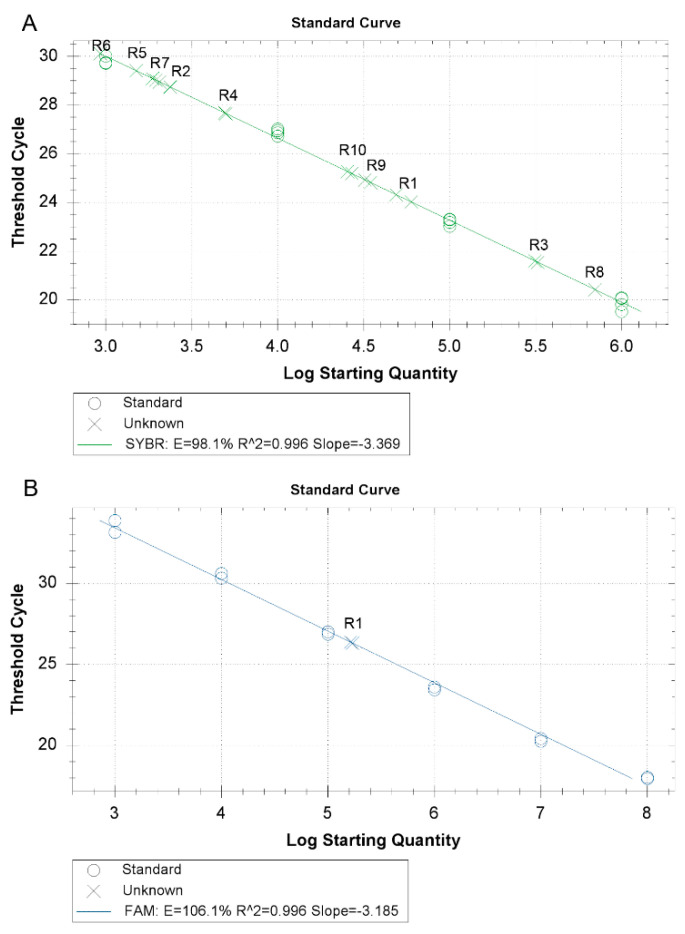
(**A**) *Rickettsia* species amplification by the new SYBR Green RT-PCR for the *ompB* gene and (**B**) the new TaqMan RT-PCR for the *ompA* gene. (R1: *R. conorii*; R2: *R. aeschlimannii*; R3: *R. massiliae*; R4: *R. raoultii*; R5: *R. monacensis*; R6: *R. helvetica*; R7: *R. slovaca*; R8: *R. felis*; R9: *Rickettsia* endosymbiont of *Haemaphysalis sulcata*; and R10: *Candidatus* Rickettsia hoogstraalii)

The reaction resulted to be positive for all the analyzed *Rickettsia* species (Figure 2A). Amplification was observed only in those samples previously selected as positive to *Rickettsia* spp. Samples positive for other tick-borne pathogens belonging to *Anaplasma*, *Ehrlichia*, *Babesia*, and *Theileria* genera resulted to be negative (Table 1).

For the exclusive amplification of *R. conorii*, a set of primers and probe was designed to selectively detect *R. conorii* DNA. The reaction was performed with the TaqMan method. The customary values obtained for the standard curve (r = 0.967 and a slope value of −3.185) are reported in Figure 2B. The analysis carried out with DNA from different *Rickettsia* species showed the presence of fluorescence only in the presence of *R. conorii* DNA. All the other *Rickettsia* species were negative as well as the samples positive for the other tick-borne pathogens (Table 1).

## 3. Discussion

The *Rickettsia* genus encompasses several species with differences in antigenic and microbiological characteristics, distribution, ecology, pathogenicity, and association with arthropod hosts [28,29]. New rickettsial syndromes have been described that are associated with the different *Rickettsia* species. In some cases, such as for *R. aeschlimannii* and *R. monacensis,* an MSF-like disease has been reported, while for other species, such as *R. raoultii* or *R. slovaca,* different clinical symptoms have been described [4]. Moreover, some species could cause severe or even lethal outcomes in subjects with predisposing conditions. For example, a fulminant course of *R. conorii israelensis* infection has been correlated with glucose-6-phosphate dehydrogenase deficiency [16,30].

In addition, a different antibiotic susceptibility among different *Rickettsia* species has been reported. Doxycycline is the treatment of choice showing the greatest efficacy against the majority of SFG *Rickettsia* species. Erythromycin and rifampicin show a variable efficacy. Rifampicin is often used when treatment with tetracycline is contraindicated, such as in children. *R. conorii* does not exhibit resistance to rifampicin, while other species such as *R. massiliae*, *R. montanensis*, *R. rhipicephali*, *R. aeschlimannii*, Catalan isolate Bar29, and *R. felis* were more resistant [28,31,32,33,34]. The rifampicin resistance observed for *R. massiliae* is probably associated with mutation of *rboB* [34].

Moreover, the availability of sensitive and reliable molecular tests is essential for conducting studies on the prevalence and endemicity of a particular species in a territory, which are essential for delimiting the areas of circulation of the species.

All these considerations explain the increasing need for sensitive diagnostic tools to identify emerging and re-emerging rickettsial infections.

Traditional molecular methods for *Rickettsia* species identification are consuming and expensive, since, for example, they are based on the amplification of pathogen DNA followed by the endonuclease fragment length polymorphism (RFLP), or a multigenic approach founded on simultaneous analysis and sequencing of many loci, such as *atpA*, *recA*, *dnaA*, *ompA*, *ompB*, and *gltA* [35].

The sensitivity of the traditional PCRs more commonly used for *Rickettsia* diagnosis is around 100 copies of rickettsial DNA protein [16,36]. Higher sensitivities are obtained for nested PCRs; however, they show numerous drawbacks, since they are time-consuming and show a higher risk of sample contamination, being two-step methods.

For this reason, in recent years several laboratories have implemented new molecular tests for the identification of *Rickettsia* species [23,24,37,38,39,40,41,42].

An RT-PCR based on the rickettsial *gltA* gene was developed that allows the amplification of DNA from members of the *Rickettsia* genus. The assay is highly sensitive (1 copy of target DNA) [24]. Afterwards, a multiplex RT-PCR assay was implemented to differentiate between scrub typhus group, typhus group, and spotted fever group rickettsiae using *47kDa*, *gltA*, and *ompB* gene targets [37]. The assay is SYBR Green-based, and the differentiation among groups is obtained by melt-curve analysis.

A pan-rickettsial RT-PCR was developed by Giulieri and collegues [38], amplifying a segment of the *16S rRNA* gene of *Rickettsia* spp. The assay is combined in a duplex RT-PCR also amplifying a *gltA* target from the TG rickettsiae. The method showed good analytical sensitivity and specificity.

Another study developed two diagnostic RT-PCRs [39]. The first is a pan-rickettsial RT-PCR targeting the *23S rRNA*, and the second is an RT-PCR specific for *R. rickettsii* species and targeting a fragment of a gene encoding the hypothetical protein A1G_04230. A sensitivity limit of 8–9 genome copies for both the assays was reported.

A specific multiplex RT-PCR assay has been developed for the detection of *R. rickettsii*, *R. parkeri*, and *R. akari* specimens [40]. These species are indigenous to the United States and are the etiologic agents of Rocky Mountain spotted fever, rickettsialpox, and *R. parkeri* rickettsiosis, respectively. In particular, the assays aimed to amplify small but specific DNA fragments of these species from a formalin-fixed, paraffin-embedded skin biopsy, overcoming the difficulty of obtaining large gene segments from formalin-fixed tissues that are usually targeted by the majority of available PCRs. Primers and probes targeted a hypothetical protein gene of the *R. rickettsii* genome and the *ompB* genes of *R. parkeri* and *R. akari*.

As regards the diagnosis of SFG *Rickettsia*, an RT-PCR was developed by Kidd and colleagues [23] for detection of SFG *Rickettsia* species in dog blood. The assay amplifies a small region of the *ompA* gene and shows a sensitivity range between 1 and 10 copies of target DNA. Species discrimination could be obtained by sequencing the smaller amplified region.

Other protocols have been developed to identify some of the new emerging *Rickettsia* species that are agents of SFGin the Mediterranean basin. In particular, a first study implemented two new TaqMan probe-based RT-PCR assays targeting the *ompA* gene for the identification of *R. aeschlimannii* and *R. africae* [41]. The assays showed high specificity and sensitivity, with the detection limit resulting to be less than 10 DNA copies.

In a second study, three RT-PCR assays targeted fragments of the *ompB* gene of the species *R. raoultii*, *R. slovaca*, and *R. aeschlimannii* [42]. The methods are based on species-specific molecular beacon or TaqMan probes and resulted to be specific and sensitive (limit of detection was at least 3 copies per reaction for all the assays).

Our work continues the efforts to improve the diagnosis of *Rickettsia* spp. The protocols developed here maintain high standards of sensitivity comparable with those of the methods reported above. In addition to what can be achieved using the methods already published, our new method also allows identification of *R. conorii*, the main agent of MSF in the Mediterranean basin, without sequencing, thus saving time and costs.

The methods described here are based on the amplification of two genes widely used in the characterization of rickettsiae. The two genes encode outer membrane proteins and contain regions highly conserved among the different species as well as hypervariable regions. In particular, the rickettsial *ompA* gene is considered an attractive target for detecting and differentiating *Rickettsia* species by molecular methods since this well-characterized gene exhibits significant variability among species [26,43]. Sequence analysis carried out in our study confirmed that the significant variability of the *ompA* and *ompB* genes makes them good targets to be used in diagnostic analyses. A region of the *ompB* gene, conserved among *Rickettsia* species, has been used to design a set of *Rickettsia* genus-specific primers. Conversely, within the *ompA* gene we identified a variable region among *Rickettsia* species, which has been used for primer and probe annealing to carry out an assay highly specific for *R. conorii*.

In conclusion, this study allowed the development of two new methods able to detect and quantify *Rickettsia* spp. pathogen DNA and also to discriminate *R. conorii* from other *Rickettsia* species (*R. aeschlimannii, R. massiliae, R. raoultii, R. monacensis, R. helvetica, R. slovaca, R. felis, Rickettsia* endosymbiont of *Haemaphysalis sulcata*, and Candidatus *Rickettsia hoogstraalii*) that are frequently detected [3,11,18,29,44,45]. Customary values obtained for the standard curve of both reactions indicate that the reactions were well-optimized. The methods reduce contamination risk and show accurate quantization of pathogen DNA and high sensitivity. Moreover, the new assays are rapid and easy to perform, and could therefore be easily implemented in laboratories with molecular facilities and added to existing molecular tools. It was reported that the development of RT-PCRs for the diagnosis of rickettsioses reduces the delay in the diagnosis of rickettsial infections and allows improvements in the efficiency of managing patients with suspected cases of rickettsiosis [46,47]. All these characteristics of the new developed methods are significant, as the rapid diagnosis of rickettsial diseases is crucial for an effective treatment of the illness.

## 4. Materials and Methods

### 4.1. Rickettsia Conorii Malish 7 Strain Culture

*R. conorii* Malish 7 strain (ATCC^®^ VR-613™) was cultured in VERO cells, a lineage of cells isolated from kidney epithelial tissue of African green monkey. Cells were maintained at 37 °C, in a humid atmosphere with 5% CO_2_, using the growth medium MEM (Minimal Essential Medium buffered-salt, Sigma, St. Louis, MO, USA), supplemented with penicillin 50 IU/mL, streptomycin 100 g/mL, amphotericin 2.5 g/mL, and fetal calf serum at 10%. The strain was used as the reference material for standardization and optimization of the RT-PCRs and to evaluate the sensitivity of the assays.

### 4.2. Field Samples for Specificity Analyses

In order to analyze the specificity of the new assays, DNA was extracted from field samples (vertebrate hosts or ticks), humans, or *R. conorii* cell cultures using the PureLink Genomic Mini kit (Applied Biosystems, Waltham, MA USA) and quantified using a Nanodrop ND1000 Spectrophotometer (NanoDrop Technologies, Wilmington, DE, USA).

DNA samples were analyzed through PCRs for *Rickettsia* spp. [48,49,50], *Anaplasma* spp. [51,52,53], and *Ehrlichia* spp. [54], and by Reverse Line Blot for *Babesia* spp. and *Theileria* spp. [55].

PCRs were performed in a reaction buffer containing 1.5 mM MgCl_2_, 0.2 mM dNTPs, 0.4 mM primers, and 0.025 U/μL of Taq polymerase (5 U/μL) (Promega, Madison, WI, USA). PCR products were visualized after electrophoresis in a 2% agarose gel with 1X SYBR Safe.

PCR products from *Rickettsia* spp. positive samples were purified by Wizard^®^ SV Gel and PCR Clean-Up System (Promega, Madison, WI, USA) and sent for sequencing (Macrogen Inc., Amsterdam, The Netherlands). Sequences analysis was performed by MEGA software [56], and comparison of obtained sequences with the ones present in Genbank was conducted using the Basic Local Alignment Search Tool (BLAST).

### 4.3. Primer Design and Selection

*OmpB* and *ompA* gene sequences from many different *Rickettsia* species (Table 2) were selected from GenBank and aligned using Clustal W in order to identify the appropriate region for primer design using the Primer Express 3.0 software.

The designed *Rickettsia* genus-specific primer sequences for SYBR Green RT-PCR are qOmpBFw (CAGCCTGACAGAACCGCTAAA) and qOmpBRev (CGATTCCGTACTCCAATCTTAGCA), targeting the *ompB* gene. The amplified fragment of the *ompB* gene was 84 bp.

**Figure 3:**
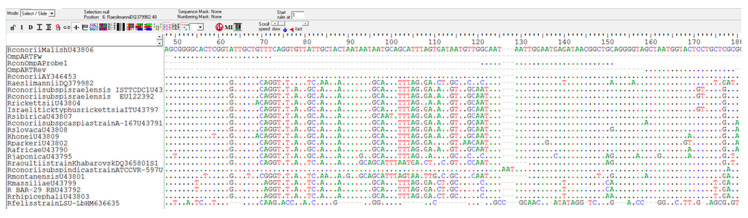
Region of the *ompA* gene in which the primers and the probe for the RT-PCR specific for *R. conorii* have been designed. The sequence of *R. conorii* in this region differs from that of other *Rickettsia* species.

**Figure 4:**
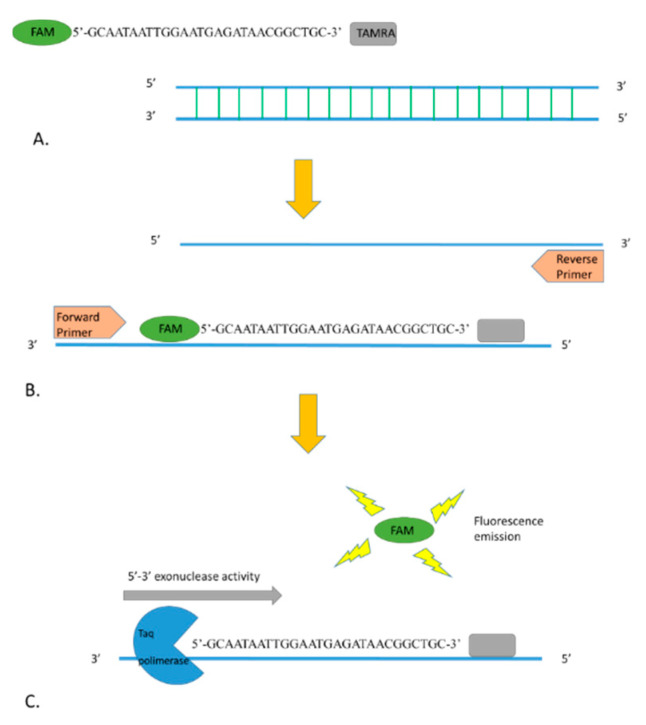
(**A**) Fluorescence of FAM fluorophore at the 5′ end of the probe is quenched by the TAMRA (quencher) present at the 3′ end. (**B**) During the annealing step of the RT-PCR, the probe is incorporated in the target sequence. (**C**) Exonuclease activity of the *Taq* polymerase during the amplification degrades the probe, and the fluorophore can release the fluorescence.

For *R. conorii*-specific RT-PCR, primers and probe were designed in a region of the *ompA* gene where differences between *R. conorii* and the other *Rickettsia* species occurred (Figure 3). The new primers are OmpARTFw (CGGGGCACTCGGTATTGCTGTTT) and OmpARTRev (GCGAGCAGGAGTACCATTAGC), and the probe is RconOmpAProbe1 5′-FAM (GCAATAATTGGAATGAGATAACGGCTGC)-3′TAMRA. The amplified fragment of the *ompA* gene was 129 bp. The quencher at the 3′ end (tetramethylrhodamine, or TAMRA) absorbs the fluorescence of the dye label (6-carboxyfluorescein, or FAM) present at the 5′ terminus of the probe. When the probe is incorporated in the target sequence and amplification occurs, the dye is removed by the 3’ to 5’ exonuclease activity of the *Taq* polymerase and it is free to release fluorescence that can be specifically detected by the instrument (Figure 4).

### 4.4. Cloning and Sequencing of PCR Products

In order to obtain the standards for the RT-PCR, a PCR with the designed primers was carried out on the DNA extracted from *R. conorii conorii* Malish 7 strain cultured in VERO cells using the conditions previously described and an annealing temperature of 60 °C. PCR products were purified, inserted into the cloning vector pCR2.1 TOPO TA (Applied Biosystems, Waltham, MA USA), and propagated in *Escherichia coli* (One Shot TOP10 Chemically Competent *E. coli,* Life Technologies, Carlsbad, CA, USA). Plasmid DNA was extracted using the Wizard purification system SV minipreps DNA purification system (Promega, Madison, WI, USA), quantified using a Nanodrop ND1000 Spectrophotometer, and sent for sequencing (Macrogen Inc., Amsterdam, The Netherlands). To obtain a series of standards covering a range from 10 to 10^8^ copies of DNA/μL, 1:10 serial dilutions were prepared; aliquots of these dilutions were stored at −20 °C and thawed only once.

### 4.5. Real-Time PCR Optimization

RT-PCRs were carried out in a CFX96 Termocycler (Biorad, Hercules, CA, USA). For the assay optimization, different annealing temperatures (58, 60, and 62 °C) and primer concentrations (0.25, 0.5, and 0.8 µM) were tested. Each reaction was performed in duplicate and in the presence of at least a no-template control.

SYBR Green RT-PCR final protocol included a final volume of 20 µL comprising 10 µL of ITaq SYBR Smx Rox (Biorad, Hercules, CA, USA), 0.5 μM of primers, and 2 μL of template DNA. The final thermal protocol comprised 95 °C for 15 s, followed by 40 cycles each comprising a step at 95 °C for 15 s and an annealing/elongation at 60 °C for 1 min.

TaqMan reactions were carried out using the previously described thermal protocol in a final volume of 20 µL comprising 10 µL of VeriQuest Probe qPCR Master Mix (Applied Biosystems, Waltham, MA, USA), 0.5 μM of primers, 0.25 μM of probe, and 2 μL of template DNA.

Fluorescence increase was detected during the polymerization phase and data were analyzed by the CFX Manager 1.6 software.

### 4.6. Specificity and Sensitivity of the New Assays

Specificity was verified both in silico using blast analysis on the GenBank database and in vitro using *R. conorii (ATCC^®^ VR-613™* cultured in VERO cells) and testing a local collection panel of DNAs belonging to different *Rickettsia* species previously identified sequencing *Rickettsia* reference genes (*ompB*, *ompA*, and *gltA*). In particular, tested samples included *R. massiliae* (KM081694, KM081702, KM115409)*, R. aeschlimannii* (KM081686, KM081699, KM115415), *R. raoultii* (KM081695)*, R. monacensis* (KM081687, KM081697, KM115411), *R. helvetica* (KM081691, KM115413), *R. slovaca* (KM081689, KM081700, KM115410)*, R. felis* (KM006784, KM006812, KM006832), *Rickettsia* endosymbiont of *Haemaphysalis sulcata* (KM081684), and *Candidatus* Rickettsia hoogstraalii (KM081685). Assay specificity was also tested in the presence of DNA from pathogens other than *Rickettsia* such as *Anaplasma*, *Ehrlichia*, *Babesia*, and *Theileria.*

Sensitivity was determined using 10-fold serial dilutions of the standards from 10^7^ to 10^-1^ DNA copies per reaction.

## Figures and Tables

**Table 1:** Results of the amplification with the two new methods in the presence of DNA from different *Rickettsia* species and from other tick-borne pathogens.

	*Rickettsia* spp. RT-PCR (*ompB*)	*R. conorii* RT-PCR (*ompA*)
*R. conorii*	+	+
*R. aeschlimannii*	+	-
*R. massiliae*	+	-
*R. raoultii*	+	-
*R. monacensis*	+	-
*R. helvetica*	+	-
*R. slovaca*	+	-
*R. felis*	+	-
*Rickettsia* endosymbiont of *Haemaphysalis sulcata*	+	-
*Candidatus* Rickettsia hoogstraalii	+	-
*Babesia bigemina*	-	-
*Babesia bovis*	-	-
*Theieleria annulata*	-	-
*Ehrlichia canis*	-	-
*Anaplasma marginale*	-	-
*Anaplasma ovis*	-	-
*Anaplasma phagocytophilum*	-	-

**Table 2:** Accession numbers of *ompB* and *ompA* gene sequences from many different *Rickettsia* species selected from GenBank and used in this study for primer and probe design and for in silico specificity analysis.

*Rickettsia* Species	*ompB* GenBank Acc. n.	*ompA* GenBank Acc. n.
*R. conorii conorii*	AF123721.1	U43806
*R. conorii* Indian tick typhus	AF123726.1	597U437
*R. conorii* subsp. Caspia	AY643093.1	167U4379
*R. conorii* Israeli tick typhus	AF123712.1	U43797
*R. massiliae*	AF123714.1	U43799
*R. raoultii*	DQ365798.1	DQ365801S1
*R. aeschlimannii*	AF123705.1	DQ379982
*R. slovaca*	AF123723.2	U43808
*R. monacensis*	EF380356.1	FJ919650
*R. helvetica*	AF123725.1	
*R. rhipicephali*	AF123719.1	U43803
*R. felis*	GQ385243	HM636635
*Candidatus* R. hoogstraalii	EF629536.1	
*Rickettsia* endosimbiont of *I. scapularis*	EF433951.1	
*R. mongolotimonae*	DQ097083.1	DQ097082.1
*R. sibirica*	AF123722.1	*U43807*
*R. africae*	AF123706.1	U43790
*R. honei*	AF123711.1	U43809
*R. rickettsii*	X16353.1	U43804
*R. montanensis*	AF123716.1	U43801
*R. parkeri*	AF123717.1	U43802
*R. japonica*	AB003681.1	U43795
*R. prowazekii*	DQ926859.1	
*R. australis*	AF123709.1	
*R. heilongjiangensis*	AY260451.1	
R BAR-29		RBU43792

Acc. n.: Accession Number.

## References

[1] Portillo A., de Sousa R., Santibáñez S., Duarte A., Oteo J.A. (2017). Guidelines for the Detection of *Rickettsia* spp.. Vector-Borne Zoonotic Dis..

[2] Merhej V., Angelakis E., Socolovschi C., Raoult D. (2014). Genotyping, evolution and epidemiological findings of *Rickettsia* species. Infect. Genet. Evol..

[3] Fernández de Mera I.G., Blanda V., Torina A., Dabaja M.F., Ali E.R., Cabezas-Cruz A., de la Fuente J. (2018). Identification and molecular characterization of spotted fever group rickettsiae in ticks collected from farm ruminants in Lebanon. Ticks Tick Borne Dis..

[4] Oteo J.A., Portillo A. (2012). Tick-borne rickettsioses in Europe. Ticks Tick Borne Dis..

[5] Dieme C., Bechah Y., Socolovschi C., Audoly G., Faye O. (2015). Transmission potential of *Rickettsia felis* infection by *Anopheles gambiae* mosquitoes. Proc. Natl. Acad. Sci. USA.

[6] Adem P.V. (2019). Emerging and re-emerging rickettsial infections. Semin Diagn Pathol..

[7] Merhej V., Raoult D. (2011). Rickettsial evolution in the light of comparative genomics. Biol. Rev. Camb. Philos. Soc..

[8] Parola P., Paddock C.D., Raoult D. (2005). Tick-Borne Rickettsioses around the World: Emerging Diseases Challenging Old Concepts. Clin. Microbiol. Rev..

[9] Parola P., Socolovschi C., Raoult D. (2009). Deciphering the relationships between *Rickettsia conorii conorii* and *Rhipicephalus sanguineus* in the ecology and epidemiology of Mediterranean spotted fever. Ann. N. Y. Acad. Sci..

[10] Duque V., Ventura C., Seixas D., da Cunha S., Duque V., Melico-Silvestre A., Barai A., Mendonca N., Martins J. (2012). Mediterranean spotted fever and encephalitis: A case report and review of the literature. J. Infect. Chemother..

[11] Blanda V., Torina A., La Russa F., D’Agostino R., Randazzo K., Scimeca S., Giudice E., Caracappa S., Cascio A., de la Fuente J. (2017). A retrospective study of the characterization of *Rickettsia* species in ticks collected from humans. Ticks Tick Borne Dis..

[12] Parola P., Socolovschi C., Jeanjean L., Bitam I., Fournier P.-E., Sotto A., Labauge P., Raoult D. (2008). Warmer weather linked to tick attack and emergence of severe rickettsioses. PLoS Negl. Trop. Dis..

[13] Torina A., Villari S., Blanda V., Vullo S., La Manna M.P., Azgomi M.S., Liberto D.D., de la Fuente J., Sireci G. (2020). Innate Immune Response to Tick-Borne Pathogens: Cellular and Molecular Mechanisms Induced in the Hosts. Int. J. Mol. Sci..

[14] Rovery C., Brouqui P., Raoult D. (2008). Questions on Mediterranean spotted fever a century after its discovery. Emerg. Infect. Dis..

[15] Raoult D., Weiller P.J., Chagnon A., Chaudet H., Gallais H., Casanova P. (1986). Mediterranean spotted fever: Clinical, laboratory and epidemiological features of 199 cases. Am. J. Trop. Med. Hyg..

[16] Giammanco G.M., Vitale G., Mansueto S., Capra G., Caleca M.P., Ammatuna P. (2005). Presence of *Rickettsia conorii subsp. israelensis*, the causative agent of Israeli spotted fever, in Sicily, Italy, ascertained in a retrospective study. J. Clin. Microbiol..

[17] Torina A., Fernández de Mera I.G., Alongi A., Mangold A.J., Blanda V., Scarlata F., Di Marco V., de la Fuente J. (2012). *Rickettsia conorii* Indian tick typhus strain and *R. slovaca* in humans, Sicily. Emerg. Infect. Dis..

[18] Cascio A., Torina A., Valenzise M., Blanda V., Camarda N., Bombaci S., Iaria C., De Luca F., Wasniewska M. (2013). Scalp eschar and neck lymphadenopathy caused by *Rickettsia massiliae*. Emerg. Infect Dis..

[19] Kernif T., Leulmi H., Raoult D., Parola P. (2016). Emerging Tick-Borne Bacterial Pathogens. Microbiol. Spectr..

[20] Ciceroni L., Pinto A., Ciarrocchi S., Ciervo A. (2006). Current knowledge of rickettsial diseases in Italy. Ann. N. Y. Acad. Sci..

[21] Gomez-Barroso D., Vescio M.F., Bella A., Ciervo A., Busani L., Rizzo C., Rezzo G., Pezzotti P. (2019). Mediterranean spotted fever rickettsiosis in Italy, 2001–2015: Spatio-temporal distribution based on hospitalization records. Ticks Tick Borne Dis..

[22] Pennisi M.G., Caprì A., Solano-Gallego L., Lombardo G., Torina A., Masucci M. (2012). Prevalence of antibodies against *Rickettsia conorii*, *Babesia canis*, *Ehrlichia canis*, and *Anaplasma phagocytophilum* antigens in dogs from the Stretto di Messina area (Italy). Ticks Tick Borne Dis..

[23] Kidd L., Maggi R., Diniz P.P.V.P., Hegarty B., Tucker M., Breitschwerdt E. (2008). Evaluation of conventional and real-time PCR assays for detection and differentiation of Spotted Fever Group *Rickettsia* in dog blood. Vet. Microbiol..

[24] Stenos J., Graves S.R., Unsworth N.B. (2005). A highly sensitive and specific real-time PCR assay for the detection of spotted fever and typhus group Rickettsiae. Am. J. Trop Med. Hyg..

[25] Segura F., Pons I., Sanfeliu I., Nogueras M.M. (2016). Shell-vial culture, coupled with real-time PCR, applied to *Rickettsia conorii* and *Rickettsia massiliae*-Bar29 detection, improving the diagnosis of the Mediterranean spotted fever. Ticks Tick Borne Dis..

[26] Fournier P.E., Dumler J.S., Greub G., Zhang J., Wu Y., Raoult D. (2003). Gene sequence-based criteria for identification of new rickettsia isolates and description of *Rickettsia heilonjiangensis* sp. Nov. J. Clin. Microbiol..

[27] Fournier P.E., Raoult D. (2007). Identification of rickettsial isolates at the species level using multi-spacer typing. BMC Microbiol..

[28] Eremeeva M.E., Bosserman E.A., Demma L.J., Zambrano M.L., Blau D.M., Dasch G.A. (2006). Isolation and identification of *Rickettsia massiliae* from *Rhipicephalus sanguineus* ticks collected in Arizona. Appl. Environ. Microbiol..

[29] Scarpulla M., Barlozzari G., Marcario A., Salvato L., Blanda V., De Liberato C., D’Agostini C., Torina A., Macri G. (2016). Molecular detection and characterization of spotted fever group rickettsiae in ticks from Central Italy. Ticks Tick Borne Dis..

[30] Regev-Yochay G., Segal E., Rubinstein E. (2000). Glucose-6-phosphate dehydrogenase deficiency: Possible determinant for a fulminant course of Israeli spotted fever. Isr. Med. Assoc. J..

[31] Beati L., Roux V., Ortuno A., Castella J., Segura Porta F., Raoult D. (1996). Phenotypic and genotypic characterization of spotted fever group rickettsiae isolated from Catalan *Rhipicephalus sanguineus* ticks. J. Clin. Microbiol..

[32] Rolain J.M., Maurin M., Vestris G., Raoult D. (1998). In vitro susceptibilities of 27 rickettsiae to 13 antimicrobials. Antimicrob. Agents Chemother..

[33] Vanrompay D., Nguyen T.L.A., Cutler S.J., Butaye P. (2018). Antimicrobial Resistance in *Chlamydiales*, *Rickettsia*, *Coxiella*, and Other Intracellular Pathogens. Microbiol. Spectr..

[34] Rolain J.M., Raoult D., Parola P. (2007). Antimicrobial susceptibility of rickettsial agents. Rickettsial Diseases.

[35] Vitorino L., Chelo I.M., Bacellar F., Zé-Zé L. (2007). Rickettsiae phylogeny: A multigenic approach. Microbiology.

[36] Leitner M., Yitzhaki S., Rzotkiewicz S., Keysary A. (2002). Polymerase chain reaction-based diagnosis of Mediterranean spotted fever in serum and tissue samples. Am. J. Trop. Med. Hyg..

[37] Paris D.H., Blacksell S.D., Stenos J., Graves S.R., Unsworth N.B., Phetsouvanh R., Newton P.N., Day N.P.J. (2008). Real-time multiplex PCR assay for detection and differentiation of rickettsiae and orientiae. Trans. R. Soc. Trop. Med. Hyg..

[38] Giulieri S., Jaton K., Cometta A., Trellu L.T., Greub G. (2012). Development of a duplex real-time PCR for the detection of *Rickettsia* spp. and typhus group rickettsia in clinical samples. FEMS Immunol. Med. Microbiol..

[39] Kato C.Y., Chung I.H., Robinson L.K., Austin A.L., Dasch G.A., Massung R.F. (2013). Assessment of real-time PCR assay for detection of *Rickettsia* spp. and *Rickettsia rickettsii* in banked clinical samples. J. Clin. Microbiol..

[40] Denison A.M., Amin B.D., Nicholson W.L., Paddock C.D. (2014). Detection of *Rickettsia rickettsii*, *Rickettsia parkeri*, and *Rickettsia akari* in skin biopsy specimens using a multiplex real-time polymerase chain reaction assay. Clin. Infect. Dis..

[41] Tomassone L., De Meneghi D., Adakal H., Rodighiero P., Pressi G., Grego E. (2016). Detection of *Rickettsia aeschlimannii* and *Rickettsia africae* in ixodid ticks from Burkina Faso and Somali Region of Ethiopia by new real-time PCR assays. Ticks Tick Borne Dis..

[42] Jiang J., You B.J., Liu E., Apte A., Yarina T.R., Myers T.E., Lee J.S., Francesconi S.C., O’Guinn M.L., Tsertsvadze N. (2012). Development of three quantitative real-time PCR assays for the detection of *Rickettsia raoultii*, *Rickettsia slovaca*, and *Rickettsia aeschlimannii* and their validation with ticks from the country of Georgia and the Republic of Azerbaijan. Ticks Tick Borne Dis..

[43] Roux V., Fournier P.E., Raoult D. (1996). Differentiation of spottedfever group Rickettsiae by sequencing and analysis of restriction fragment length polymorphism of PCR-amplified DNA of the gene encoding the protein rOmpA. J. Clin. Microbiol..

[44] Giudice E., Di Pietro S., Alaimo A., Blanda V., Lelli R., Francaviglia F., Caracappa S., Torina A. (2014). A molecular survey of *Rickettsia felis* in fleas from cats and dogs in Sicily (Southern Italy). PLoS ONE.

[45] Parola P., Paddock C.D., Socolovschi C., Labruna M.B., Mediannikov O., Kernif T., Abdad M.Y., Stenos J., Bitam I., Fournier P.-E. (2013). Update on tick-borne rickettsioses around the world: A geographic approach [published correction appears in Clin Microbiol Rev. 2014 Jan;27(1):166]. Clin. Microbiol. Rev..

[46] Holland C.A., Kiechle F.L. (2005). Point-of-care molecular diagnostic systems-past, present and future. Curr. Opin. Microbiol..

[47] Renvoisé A., Rolain J.M., Socolovschi C., Raoult D. (2012). Widespread use of real-time PCR for rickettsial diagnosis. FEMS Immunol. Med. Microbiol..

[48] Oteo J.A., Portillo A., Santibáñez S., Blanco J.R., Pérez-Martínez L., Ibarra V. (2006). Cluster of cases of human *Rickettsia felis* infection from Southern Europe (Spain) diagnosed by PCR. J. Clin. Microbiol..

[49] Choi Y.J., Lee S.H., Park K.H., Koh Y.S., Lee K.H., Baik H.S., Choi M.S., Kim I.S., Jang W.J. (2005). Evaluation of PCR-based assay for diagnosis of spotted fever group rickettsiosis in human serum samples. Clin. Diagn. Lab. Immunol..

[50] Roux V., Rydkina E., Eremeeva M., Raoult D. (1997). Citrate synthase gene comparison, a new tool for phylogenetic analysis, and its application for the rickettsiae. Int. J. Syst. Bacteriol..

[51] Torina A., Agnone A., Blanda V., Alongi A., D’Agostino R., Caracappa S., Marino A.M.F., Marco V.D., La Fuente J. (2012). Development and validation of two PCR tests for the detection of and differentiation between *Anaplasma ovis* and *Anaplasma marginale*. Ticks Tick Borne Dis..

[52] Naranjo V., Ruiz-Fons F., Höfle U., De Mera I.G.F., Villanua D., Almazan C., Torina A., Caracappa S., Kocan K.M., Gortazar C. (2006). Molecular epidemiology of human and bovine anaplasmosis in southern Europe. Ann. N. Y. Acad. Sci..

[53] Stuen S., Nevland S., Moum T. (2003). Fatal cases of Tick-borne fever (TBF) in sheep caused by several 16S rRNA gene variants of *Anaplasma phagocytophilum*. Ann. N. Y. Acad. Sci..

[54] Munderloh U.G., Madigan J.E., Dumler J.S., Goodman J.L., Hayes S.F., Barlough J.E., Nelson C.M., Kurtti T.J. (1996). Isolation of the equine granulocytic ehrlichiosis agent, *Ehrlichia equi*, in tick cell culture. J. Clin. Microbiol..

[55] Gubbels J.M., de Vos A.P., van der Weide M., Viseras J., Schouls L.M., De Vries E., Jongejan F. (1999). Simultaneous detection of bovine *Theileria* and *Babesia* species by reverse line blot hybridization. J. Clin. Microbiol..

[56] Kumar S., Nei M., Dudley J., Tamura K. (2008). MEGA: A biologist-centric software for evolutionary analysis of DNA and protein sequences. Brief. Bioinform..

